# Myomodulation with Facial Fillers: A Comprehensive Technical Guide and Retrospective Case Series

**DOI:** 10.1007/s00266-022-03193-y

**Published:** 2022-11-28

**Authors:** Daniel Dal’Asta Coimbra, Betina Stefanello

**Affiliations:** Instituto de Dermatologia Professor Rubem David Azulay Santa Casa de Misericórdia/IDPRDA, Rio de Janeiro, Brazil

**Keywords:** Myomodulation, Hyaluronic acid, Facial aging, Dermal fillers, Mimetic muscles

## Abstract

**Background:**

Placement of fillers in close proximity to the mimetic or sphincter muscles of the face appears to enhance or suppress muscle action in a relatively predictable way.

**Methods:**

From June 2016 to June 2021, patients who underwent the first author’s technique of myomodulation with dermal fillers to address aesthetic concerns or to manage facial spasms or synkinesis were evaluated in a retrospective case series. Additionally, the authors provide a technical guide for a whole-face approach to treatment with fillers and a conceptual map for treatment of each facial subunit with a focus on myomodulation.

**Results:**

A total of 1352 patients (1108 women, 244 men; mean age, 51 years) underwent at least 1 treatment session during the 5-year study period. The treatment patterns of the study population and details of 2 representative cases are presented.

**Conclusions:**

Although not well understood mechanistically, myomodulation with injectable fillers shows promise for significant and reliable results of facial rejuvenation.

**Level of Evidence IV:**

This journal requires that authors assign a level of evidence to each article. For a full description of these Evidence-Based Medicine ratings, please refer to the Table of Contents or the online Instructions to Authors www.springer.com/00266.

**Supplementary Information:**

The online version contains supplementary material available at 10.1007/s00266-022-03193-y.

## Introduction

The applications of dermal fillers have expanded from management of static rhytids and volume restoration to intentionally influencing the balance and contractility of the facial musculature [1]. Myomodulation entails the strategic placement of dermal filler in the vicinity of the facial mimetic or sphincter muscles to either facilitate or hinder muscle action [1–3]. The theoretical basis of myomodulation arises, in part, from magnetic resonance imaging findings in young patients, which demonstrated that deep fat compartments of the face produce convexity in the overlying levator muscles [4]. This convexity is lost as fat and bone regress with aging, resulting in straightening of the levator muscles and greater influence of the opposing depressors [1, 3]. Accordingly, a bolus of filler placed beneath the muscle near its origin (e.g., in the malar region) could substitute for the lost structural support from adipose and osseous tissues. The filler acts as a fulcrum, recreating the convexity and tension of the muscle and thus increasing its contractility or restoring the tonus at rest [1, 3]. Conversely, muscle action could be restrained by filler placement superficial to the muscle in the subcutaneous layer (e.g., in the mentolabial angle) or intramuscularly (e.g., in the sphincter muscles of the periorbital or perioral regions). Muscle action also could be blunted by injecting filler near its skin insertion [1]. For instance, the nasolabial fold could be treated in this way to reduce the effect of the levator labii superioris (LLS), levator labii superioris alaeque nasalis (LLSAN), and zygomaticus major and minor.

**Fig 1:**
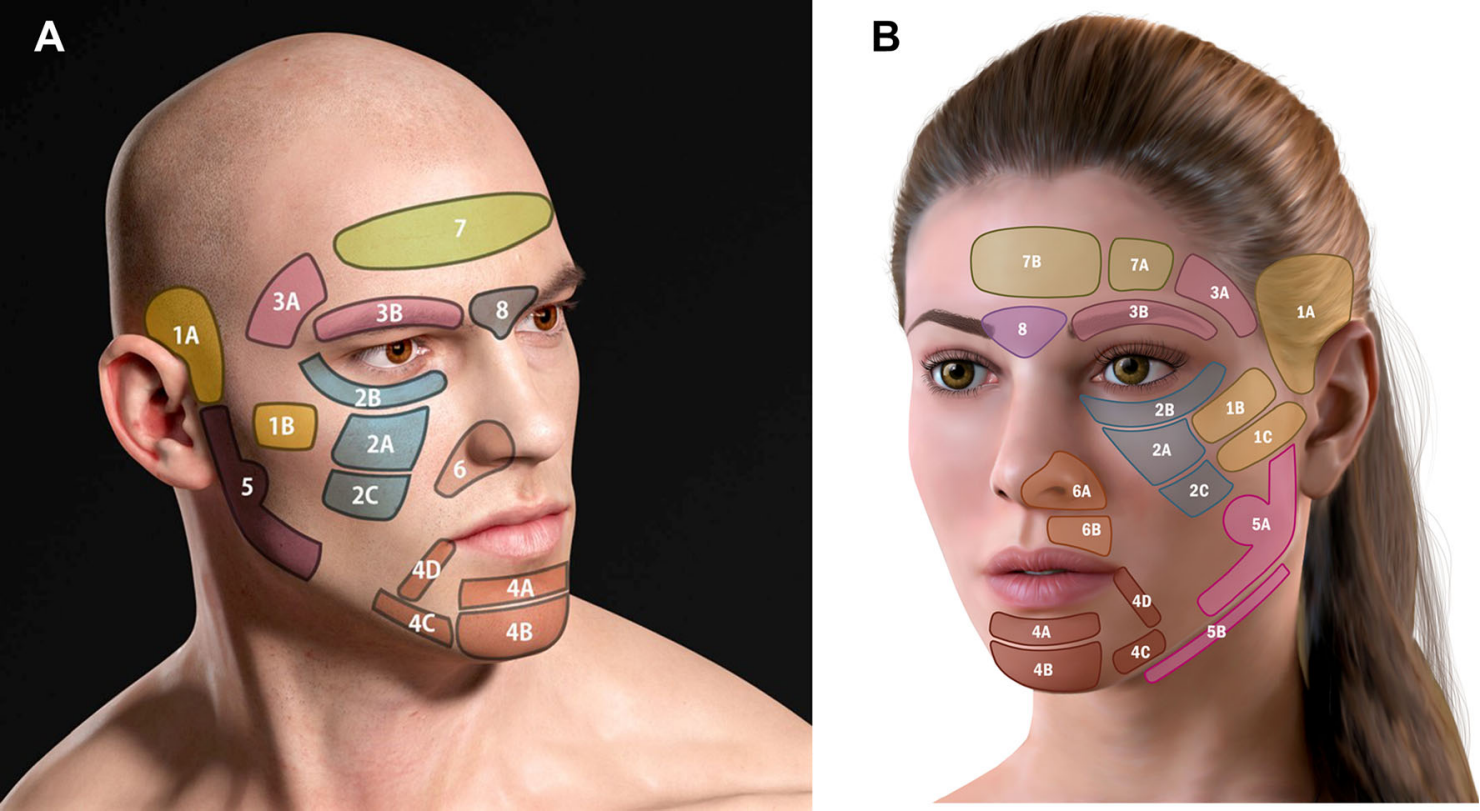
The authors’ designation of the facial subunits in men **A** and in women **B**.

Isolated placement of fillers in the supra-auricular area (i.e., the superior temple) has been shown to affect muscle contraction in nonadjacent areas–for instance, producing a broader smile with greater exposure of the lateral teeth [5]. For more than a decade, the first author has been employing the strategy of myomodulation with facial fillers in clinical practice. This work has yielded empirical findings that are consistent with the hypothetical mechanisms described above. These observations led to the development of a novel technique–the 3-Dimensional Dynamic Lift™ (3DD Lift)–that conceptualizes the face into 8 major treatment areas and smaller aesthetic subunits (Figure 1). The 3DD Lift technique involves injection of dermal fillers in a predetermined as-needed sequence, establishing the structural foundation of the midface first, then contouring the upper and lower face, and finally refining the periorbital, nasolabial, and perioral areas. Excellent aesthetic and functional results have been achieved with this technique, not only in patients seeking facial rejuvenation, but also in those with facial paralysis sequelae (e.g., synkinesis, muscular spasms).

## Methods

A retrospective case series was undertaken on patients treated with hyaluronic acid (HA)-based injectable fillers at a single cosmetic dermatology clinic in Brazil from June 2016 to June 2021. Patient medical records were reviewed for demographic variables and details about filler injection sessions. The first author performed all treatments.

Microsoft Excel (2016) was used to generate descriptive statistics and summary tables. A complete case approach was undertaken for the assessment of all treatment sessions, such that sessions with missing data on the facial subunit(s) treated were excluded from the analysis.

### Technical Guide to Filler Placement

Applying 3DD Lift, the face is conceptualized as 8 anatomic areas, with some areas further subdivided. The delineation of the facial subunits follows from the distinct maneuvers needed in these regions to effect myomodulation with injectable fillers. Treatment of every subunit is not usually necessary; however, the sequential placement of filler from the most lateral regions of the face inward to the medial areas is crucial. The authors recommend beginning treatment in the upper third of the face (i.e., the superior temple), followed by the middle third and then the lower third (as needed), always utilizing a lateral-to-medial order of injection.

This approach is consistent with the work of Casabona et al,[6]who described an injection technique in which the face is divided into anatomic regions located anteriorly and laterally to the line of ligaments (i.e., the transverse facial septum). In the anterior face, structures related to animation and expression predominate, whereas in the lateral region, muscles related to mastication stand out. Injections of dermal fillers anterior to the line of ligaments were found to have a more volumizing effect, whereas treatments lateral to this line tended to produce more lift [6]. The 3DD Lift technique prioritizes the latter. The areas located lateral to the line of ligaments are treated first to provide structural support to the face; this decreases the need for filler material anterior to the line of ligaments. This sequential approach helps avoid an overfilled appearance (i.e., pillow face or chipmunk cheeks). [6]

**Fig 2:**
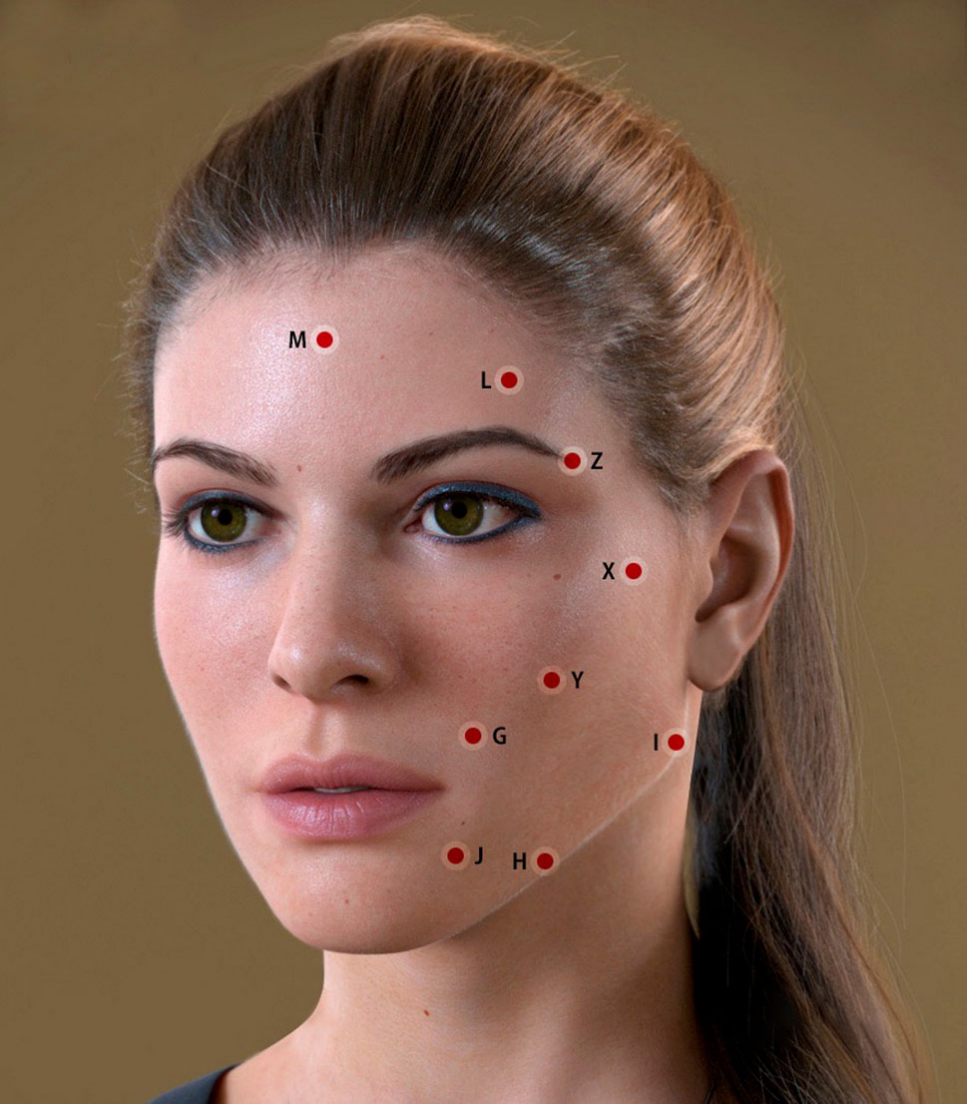
Depiction of cannula entry points for placement of filler.

The following sections provide a comprehensive technical guide to 3DD Lift. The cannula entry points referred to throughout these sections are depicted in Figure 2.

#### 1A, 1B, and 1C: The Supra-auricular (Superior Temple), Zygomatic, and Infrazygomatic Areas

Area 1A comprises the temporal hollow; that is, the superficial superior-temporal fat compartment extending downward to the tragus. The temporal hollow is typically covered by hair but can be felt manually. Treatment in this area provides structural support to the lateral region of the face. Cannula entry (21G–25G) is at point X (above the zygomatic bone) or at point I. For men, who generally have more hollowness in the area immediately above the tragus, both points of entry are utilized to provide additional structural support. The cannula proceeds superficially along the subcutaneous plane to area 1A, superior to the inferior temporal septum. Boluses of HA-based filler, ranging from 0.1 mL to 0.3 mL, are injected into the area to restore volume, with each side receiving approximately 0.6 mL to 1.2 mL of product. As the cannula is advanced in a fanning pattern, care should be given to avoid breaching the septum that divides the upper and lower temporal fat compartments. Additionally, extreme caution is needed to avoid the superficial temporal artery and the frontotemporal branch of the facial nerve.

Area 1B may be treated by means of a cannula or a needle; the latter facilitates sharper definition and more structural support. At each point above the zygomatic bone, approximately 0.1 mL to 0.2 mL is delivered, with a 90-degree injection angle. When the skin envelope is thin, entry of a cannula at point Y is recommended, with filler deposited above the periosteum. The total volume injected ranges from 0.3 mL to 0.7 mL per side.

A cannula is recommended for treatment of area 1C, with entry at point Y or I and filler injected into the superficial subcutaneous layer. Filler placed in this area provides structural support to the lateral face and creates the appearance of a slimmer lower contour. Injection volumes range from 0.3 mL to 1.0 mL per side.

#### 2A, 2B, and 2C: The Malar, Infraorbital, and Inframalar Areas

Area 2A is addressed by filler injection into the deep supraperiosteal layer or the superficial fat compartment. For supraperiosteal treatment, a sharp needle is used to deliver small (0.2–0.5 mL) boluses lateral to the infraorbital foramen. This technique supports the suborbicularis oculi fat (SOOF). Between the midpupillary line and the nose, a cannula should be used rather than a needle to avoid intravascular injection. Entry is made at point Y, the cannula tip is advanced to the uppermost part of the maxillary bone, and filler is placed in the supraperiosteal layer near the infraorbital rim. With this technique, the filler acts as a fulcrum, recreating convexity of the muscle and enhancing contractility. A second single injection into the inferolateral part of the maxillary bone may be made if greater anterior projection is desired below the zygomaticus major. If lower-eyelid bags are being treated in the same session, filler injections into the superficial fat compartments may be made as well, but a low-HA concentration, low-cohesivity filler should be chosen to avoid undesirable tension in this area. Injection volumes vary from 0.3 mL to 0.7 mL per side.

Because of thin skin and the adjacent orbital fat pads, management of the tear trough deformity with fillers is technically challenging. For treatment of area 2B, entry point Y may be used or another point closer to this area. Here, the authors recommend placing a low G-prime filler under the muscle at points along the inferior and lateral orbital grooves.

In the inframalar region (area 2C), filler placement can yield an unfavorable puffy look and impede muscle action. Instead, placement of a collagen biostimulator (e.g., poly-L-lactic acid, calcium hydroxyapatite) is advised. [7]

#### 3A and 3B: The Inferior Temple and Eyebrow Arch

Areas 3A and 3B comprise the superior orbital rim and lateral orbital rim extending to the temporal hairline. This area should be treated with extreme caution to avoid the temporal branch of the facial nerve and the temporal vessels as well as the supraorbital and supratrochlear nerves, arteries, and veins.

In area 3A, a needle is used to deposit filler, very slowly, into the deep supraperiosteal layer. Boluses of approximately 0.5 mL to 1.0 mL are deposited on each side. To ensure no filler is deposited in the deep temporal vessels, aspiration must be performed before the injection. Treatment of this area can also be performed with a cannula, with filler placed in the superficial subcutaneous layer or interfascial plane through entry point X or L. This approach requires caution because the superficial temporal vessels are located between the two laminae of the superficial temporal fascia. In addition to potential vascular complications, unfavorable outcomes of skin irregularities or vessel engorgement may occur as a result of filler placement in these planes. Therefore, we recommend superficial injections of collagen biostimulators for this area. In the first author’s clinical practice, excellent results have been achieved with a combination of HA fillers in the deep supraperiosteal layer and collagen-stimulating fillers in the superficial subcutaneous layer.

To treat area 3B, entry of a 50 mm, 22G to 25G cannula is made at the tail of the eyebrow (point Z) and is advanced along the arch of the eyebrow toward the glabella using a slow and gentle anterograde technique. The filler material is deposited in small droplets along the extension of the brow (supraorbital rim), with slightly larger volumes placed in the retro-orbicularis oculi fat (ROOF) below the tail of the brow. The total filler volume for area 3B ranges from 0.2 mL to 0.7 mL per side. In area 3B, filler must be placed in the subcutaneous layer or beneath the muscle and must not be performed with a needle because of the risk of blindness or a cerebral vascular incident secondary to intravascular infiltration into the supraorbital or supratrochlear vessels. The branches of these vessels emerge from the corresponding foramens and run toward the forehead close to the bone for approximately 1.5 cm. Above this, the supraorbital vessels proceed more superficially, and filler placement must be under the muscle in the deep supraperiosteal plane. (This approach also applies to area 7A. When there is an indication to treat both 3B and 7A in the same session, these subunits can be accessed from the same cannula entry point, Z.)

If decreased action of the upper half of the orbicularis oculi is needed, the upper eyelids also may be treated through the same cannula entry point (Z). Using a low G-prime product, small volumes of filler (<0.2 mL) are injected above the orbital septum. Indications for this include blepharospasm, synkinesis associated with facial nerve paralysis, or the sunken appearance referred to as the A-frame deformity.

#### 4A, 4B, 4C, 4D: The Mentolabial Angle, Mentum, Prejowl, and Marionette Lines

The subunits of area 4 extend from the lower lip and the anterior portion of the mandible and are laterally bounded by the depressor anguli oris (DAO). Vulnerable structures in this area include the inferior labial artery, the marginal mandibular nerve, the mental nerve, and vessels that emerge from the mental foramen.

Area 4A can be managed when restriction of the depressor labii inferioris (DLI) and mentalis is desired. To achieve this, a 21G to 25G cannula is inserted through point G, and filler is applied into the subcutaneous layer or intramuscularly, in the medial portion between the paired DLI. Alternatively, filler can be placed to augment volume in the lateral portion of 4A under the DLI by entering through point H; this maneuver is less likely to yield visible lumps on animation. Some patients also require treatment of the region above the DLI. For these cases, the authors recommend using low-to-moderate G-prime products, with access to this area achieved through entry points G or H. The total amount of filler injected ranges from 0.3 mL to 1.0 mL per side.

Area 4B can also be treated through entry point G by moving the cannula toward the chin apex while depositing small boluses of filler. Alternatively, a needle may be employed, with a 90-degree injection angle advancing toward the bone. It is necessary to aspirate before the injection. For either approach, boluses typically range from 0.1 mL to 0.3 mL. A third option for treatment of this area with a needle involves proceeding cranially from the chin apex toward the pogonion, parallel to the bone. Aspiration prior to needle injection is always essential, and it can be difficult with this technique because the needle tip does not touch the bone. Filler can also be placed in the lateral portions of 4B when the aim is to enhance chin width (more common in male patients). A total volume of 0.3 mL to 1.0 mL per side is typical.

Areas 4C and 4D can be addressed through cannula entry point H. In 4C, filler is placed subcutaneously or in the periosteum along the body of the mandible, in the transition between the face and neck, and anterior to the retaining ligament of the mandible. For area 4D, the filler is injected into the subcutaneous layer between the DLI and the DAO. Moderate to high G-prime fillers are recommended for these areas, and volumes up to 1.0 mL per side in 4C and 0.5 mL per side in 4D are typically required. The entire filler volume deposited in the mentum typically ranges from 2.0 mL to 5.0 mL, with men usually receiving larger volumes. Treatment of the lips can play a significant role in the myomodulation of the lower third of the face; however, the techniques to manage this area are intensive and were considered outside the scope of this description of 3DD Lift.

#### 5A and 5B: The Angle and Body of the Mandible and Inframandibular Areas

Area 5A comprises the angle and body of the mandible and is bounded by the tragus. At-risk structures in this area are the parotid glands and ducts and the masseter. Area 5B includes the inframandibular subunit in the neck close to the inferior-internal border of the mandible. Here, caution must be given to avoid the facial artery, the submandibular glands, and the marginal mandibular nerve.

Area 5A can be treated with a needle when the aim is to enhance projection of the mandibular angle. A single bolus of 0.3 mL to 0.5 mL is deposited in the angle of the mandible close to the bone. More than one bolus may be required to achieve the desired outcome. Alternatively, a cannula can be inserted through entry point I or H, and filler can be placed subcutaneously along the entire mandible (ie, body, angle, and ramus) to enhance definition and increase the intergonial distance. For elongation of the jawline, filler can be injected into the subcutaneous plane, posterior to the angle of the mandible. A high or very high G-prime filler is applied, and typical volumes differ by gender: 0.5 mL to 1.0 mL per side in women versus 1.0 mL to 3.0 mL per side in men. The authors prefer this alternative technique because it allows for definition of the jawline with a lower volume of filler and because filler placement in the subcutaneous plane of area 5A appears to have a greater myomodulatory effect than does placement near the bone. It is speculated that subcutaneous filler placement in 5A creates a mechanical obstacle for the platysma on animation.

Area 5B may be managed when the aims are to improve the contour of the lower face and to address sagging neck skin without widening the jawbone. Treatment of this region is only indicated when the underlying bony structure of the mandible is defined and there are no fat deposits in the inframandibular area below the jawline. A cannula is inserted through entry point I, and filler is placed subcutaneously in the inferior-internal border of the mandible. The total volume delivered ranges from 0.2 mL to 0.5 mL per side.

#### 6A and 6B: The Central Face and Supraperioral Areas

Area 6A is a triangular subunit on the maxilla that comprises the pyriform fossa, the upper portions of the nasolabial folds, and the suprabuccal region. The superior labial artery, which supplies the nasal ala, columella, and vestibulum, is a sensitive structure in this area. Depending on the desired result, a cannula or a needle may be used to manage area 6A. To restore convexity to the midface (owing to bone resorption with aging or to congenital skeletal abnormalities), a single bolus of 0.2 mL to 0.5 mL per side may be injected through a needle into the supraperiosteal plane of the pyriform fossa. Before the injection, the tip of the needle should gently touch the bone and be aspirated.

When the aim is to restrict the activity of the depressor septi nasi (DSNM), the LLS (ie, the muscles associated with smiling), and the LLSAN, a cannula is inserted through entry point G into the subcutaneous layer, and filler is deposited throughout area 6A. This maneuver is commonly employed to treat a high smile line (ie, gummy smile).

For treatment of area 6B (supraperioral), a 25G to 27G cannula is inserted through entry point G into the lower portion of the nasolabial fold, and filler is deposited above the muscle or into the muscle to restrict the contractile force of the orbicularis oris. HA fillers with low to moderate G primes are recommended for this region, and the typical volume is 0.2 mL to 0.5 mL per side. The nasolabial folds may be addressed with superficial injections into the dermis or subcutaneous layer using a needle or cannula through entry point G.

#### 7A and 7B: The Forehead

Area 7 corresponds to the forehead concavity that extends from one temporal crest to the other and from the upper hairline to the brows. In men, the forehead is regarded as a single area. In women, the forehead comprises subunits 7A and 7B, and filler placement usually is limited to 7A. This distinction is made because, in women, the forehead concavity is more pronounced lateral to the midpupillary lines. The frontal branch of the superficial temporal artery and the supraorbital and supratrochlear vessels are vulnerable structures located in the superficial planes of the forehead.

In women, area 7A is often treated with a moderate G-prime product, with volumes ranging from 0.1 mL to 0.3 mL per side. This area is commonly managed in conjunction with area 3B and (as discussed previously) is accessed through entry point Z. In men, the entire forehead is addressed by means of a 21G to 25G cannula inserted through entry point L, and filler volumes range from 0.5 mL to 1.0 mL per side. A moderate-to-high G-prime filler is placed under the muscle to increase forehead convexity and restore the resting tonus of the frontalis, thereby reducing hyperkinetic lines in the forehead.

#### 8: The Glabella

Area 8 corresponds to the glabella, which is affected by the depressor supercilii, the medial corrugator supercilii, and the procerus. Sensitive structures in this area include the supratrochlear vessels. The vessels that emerge from the supratrochlear foramen run deep for approximately 1.5 cm and then travel superficially toward the forehead.

To manage this area, a 22G to 25G blunt-tipped cannula is inserted through entry point M and advanced parallel to the supratrochlear vessels. To avoid vascular cannulation, filler material should only be placed in the subcutaneous layer, and cannula advancement should be slow and gentle. The area also may be accessed through entry point Z, using a 50-mm cannula. Typical treatment volumes range from 0.1 mL to 0.5 mL. Given the high risk of intravascular injection, this area should only be managed by an experienced practitioner.

## Results

A total of 1352 adults (1108 women, 82%; 244 men, 18%) received 3DD Lift myomodulation treatment (Table 1) with HA-based fillers, including Voluma™, Volbella™, Volift™, or Volux™ (all, Juvéderm; Allergan, Inc.). Some of the patients underwent multiple treatment sessions in the 5-year study period (Table 2). The mean patient age at first myomodulation treatment was 51 years (men, 47 years, range, 21-78 years; women, 52 years, range, 20-94 years). Older patients generally received more treatment sessions.

**Table 1:** Distribution of Patients by Gender and Age Range at First Myomodulation Session

Age Range, years	No. of Men	Percentage	No. of Women	Percentage	Full Study Population	Percentage
20 - 24	3	1.2	6	0.5	9	0.7
25 - 29	6	2.5	19	1.7	25	1.8
30 - 34	30	12.3	54	4.9	84	6.2
35 - 39	45	18.4	115	10.4	160	11.8
40 - 44	41	16.8	162	14.6	203	15.0
45 - 49	26	10.7	148	13.4	174	12.9
50 - 54	28	11.5	134	12.1	162	12.0
55 - 59	19	7.8	127	11.5	146	10.8
60 - 64	25	10.2	123	11.1	148	10.9
65 - 69	10	4.1	102	9.2	112	8.3
70 - 74	8	3.3	66	6.0	74	5.5
75 - 79	3	1.2	27	2.4	30	2.2
80 and older	0	0.0	25	2.3	25	1.8
Total	244	100.0	1108	100.0	1352	100.0

**Table 2:** Distribution of patients by mean number of myomodulation sessions and mean product volume received (June 2016 to June 2021)

	Men				Women				Full Study Population			
Age group at first treatment, year	Total	%	Mean no. of treatment sessions	Mean volume, mL	Total	%	Mean no. of treatment sessions	Mean volume, mL	Total	%	Mean no. of treatment sessions	Mean volume, mL
20 - 24	11	0.8	4	1.8	23	0.3%	4	1.0	34	0.4%	4	1.3
25 - 29	40	2.8	7	1.5	58	0.8%	3	1.2	98	1.1%	4	1.3
30 - 34	125	8.8	4	1.6	209	2.8%	4	1.1	334	3.8%	4	1.3
35 - 39	181	1.7	4	1.3	499	6.7%	4	1.0	680	7.6%	4	1.1
40 - 44	286	2.1	7	1.4	963	1.9%	6	1.1	1,249	14.0%	6	1.2
45 - 49	152	1.7	6	1.3	1,097	1.7%	7	1.1	1,249	14.0%	7	1.1
50 - 54	195	13.7	7	1.3	1,086	1.5%	8	1.1	1,281	14.4%	8	1.1
55 - 59	138	9.7	7	1.2	985	1.2%	8	1.1	1,123	12.6%	8	1.1
60 - 64	162	1.4	6	1.2	925	12.4%	8	1.1	1,087	1.2%	7	1.1
65 - 69	60	4.2	6	1.4	659	8.8%	6	1.1	719	8.1%	6	1.1
70 - 74	59	4.1	7	1.2	551	7.4%	8	1.1	610	6.9%	8	1.1
75 - 79	15	1.1	5	1.6	229	3.1%	8	1.0	244	2.7%	8	1.1
80 and older	0	0.0	0	NA	184	2.5%	7	0.9	184	2.1%	7	0.9
Total	1424	100.0	6	1.3	7468	100.0	7	1.1	8892	100.0	7	1.1

Of the 9355 treatment sessions performed, 463 sessions (5.0%) were missing data on the areas treated and were excluded from the analyses. The total number of included sessions was 8892; 7468 of these were performed in women, and 1424 were in men (Table 2). The per-patient mean number of treatment sessions received was 7 for women and 6 for men, with a mean total injection volume of 1.1 mL per side and 1.3 mL per side, respectively.

The most commonly treated areas of the face were those lateral to the line of ligaments, constituting subunits 1A+1B+1C+3A+5A; these accounted for 53% of the total number of treatment sessions (Table 3). Subunit 1A was the most frequent site of treatment (68.7% of patients; 70.4% women and 61.1% men) and was commonly re-treated in patients who underwent multiple sessions (data not shown).

**Table 3.** Distribution of treatment session by facial subunit addressed and mean volume of filler injected

	Men			Women			Total		
Facial subunit(s) treated	No. of sessions	%	Mean volume, mL	No. of sessions	%	Mean volume, mL	No. of sessions	%	Mean volume, mL
Total	1424	100.0		7468	100.0		8892	100.0	
1A	179	12.6	1.6	946	12.7	1.6	1125	12.7	1.6
1B	177	12.4	0.8	1103	14.8	0.9	1280	14.4	0.9
1C	27	1.9	0.9	391	5.2	0.9	418	4.7	0.9
1A+1B+1C	383	26.9	1.2	2440	32.7	1.2	2823	31.7	1.2
2A	198	13.9	1.2	868	11.6	0.9	1066	12.0	1.0
2B	97	6.8	1.0	417	5.6	0.9	514	5.8	0.9
2C	10	0.7	0.9	68	0.9	0.7	78	0.9	0.7
2A+2B+2C	305	21.4	1.1	1353	18.1	0.9	1658	18.6	0.9
3A	105	7.4	1.4	472	6.3	1.2	577	6.5	1.3
3B	111	7.8	1.4	428	5.7	0.9	539	6.1	1.0
3A+3B	216	15.2	1.4	900	12.1	1.1	1116	12.6	1.1
4A	61	4.3	1.0	330	4.4	0.9	391	4.4	0.9
4B	52	3.7	1.8	220	2.9	1.7	272	3.1	1.7
4C	85	6.0	1.4	265	3.5	0.8	350	3.9	0.9
4D	45	3.2	0.8	396	5.3	0.8	441	5.0	0.8
4A+4B+4C+4D	243	17.1	1.3	1211	16.2	1.0	1454	16.4	1.0
5A	230	16.2	2.1	1040	13.9	1.5	1270	14.3	1.6
5B	0	0.0	0	3	0.0	1.1	3	0.0	1.1
5A+5B	230	16.2	2.1	1043	14.0	1.5	1273	14.3	1.6
6A	7	0.5	1.0	49	0.7	1.0	56	0.6	1.0
6B	5	0.4	0.2	298	4.0	0.5	303	3.4	0.5
6A+6B	12	0.8	0.7	347	4.6	0.6	359	4.0	0.6
7A	20	1.4	0.5	124	1.7	0.3	144	1.6	0.3
7B	3	0.2	2.0	4	0.1	0.6	7	0.1	1.2
7A+7B	23	1.6	0.6	128	1.7	0.3	151	1.7	0.3
8	12	0.8	0.7	46	0.6	0.6	58	0.7	0.6
Lateral areas, line of ligaments (1A+1B+1C+3A+5A)	718	50.4	1.5	3952	52.9	1.3	4670	52.5	1.3

Myomodulatory treatments categorized by injection device and brand of HA product are summarized in Table 4. Voluma™ was used most frequently, followed by Volift™, Volbella™, and Volux™. Most treatments (81%) were performed with a cannula.

**Table 4:** Distribution of patients by injection device and brand of filler type received

Variable	Men	%	Women	%	Combined	%
Total no. of treatment sessions during the study period, with any filler produce	1442	16.0	7556	84.0	8998^a^	100.0
No. injected by needle	205	14.2	1128	14.9	1333	14.8
No. delivered by cannula	1180	81.8	6099	80.7	7279	80.9
Volbella™, no. of treatments	79	5.5	398	5.3	477	5.3
Total volume, mL	74.4		320.4		394.8	
Mean volume per treatment, mL	0.9		0.8		0.8	
Voluma™, no. of treatments	1167	80.9	5846	77.4	7013	77.9
Total volume, mL	1631.7		6749.4		8381.1	
Mean volume per treatment, mL	1.4		1.2		1.2	
Volift™, no. of treatments	141	9.8	1157	15.3	1298	14.4
Total volume, mL	112.1		815.1		927.2	
Mean volume per treatment, mL	0.8		0.7		0.7	
Volux™, no. of treatments	29	2.0	97	1.3	126	1.4
Total volume, mL	34.0		88.7		122.7	
Mean volume per treatment, mL	1.2		0.9		1.0	
Other product, no. of treatments	26	1.8	58	0.8	84	0.9
Total volume, mL	24.6		50.5		75.1	
Mean volume per treatment, mL	0.9		0.9		0.9	

Voluma™, Volbella™, Volift™, and Volux™ are manufactured by Juvéderm.

^a^The 106-session discrepancy (8998 vs. 8892) in Tables 2 and 3 versus Table 4 is owing to data missing from the sample. These missing data were omitted from the complete-case statistical analysis.

### Case Presentations

**Fig 3:**
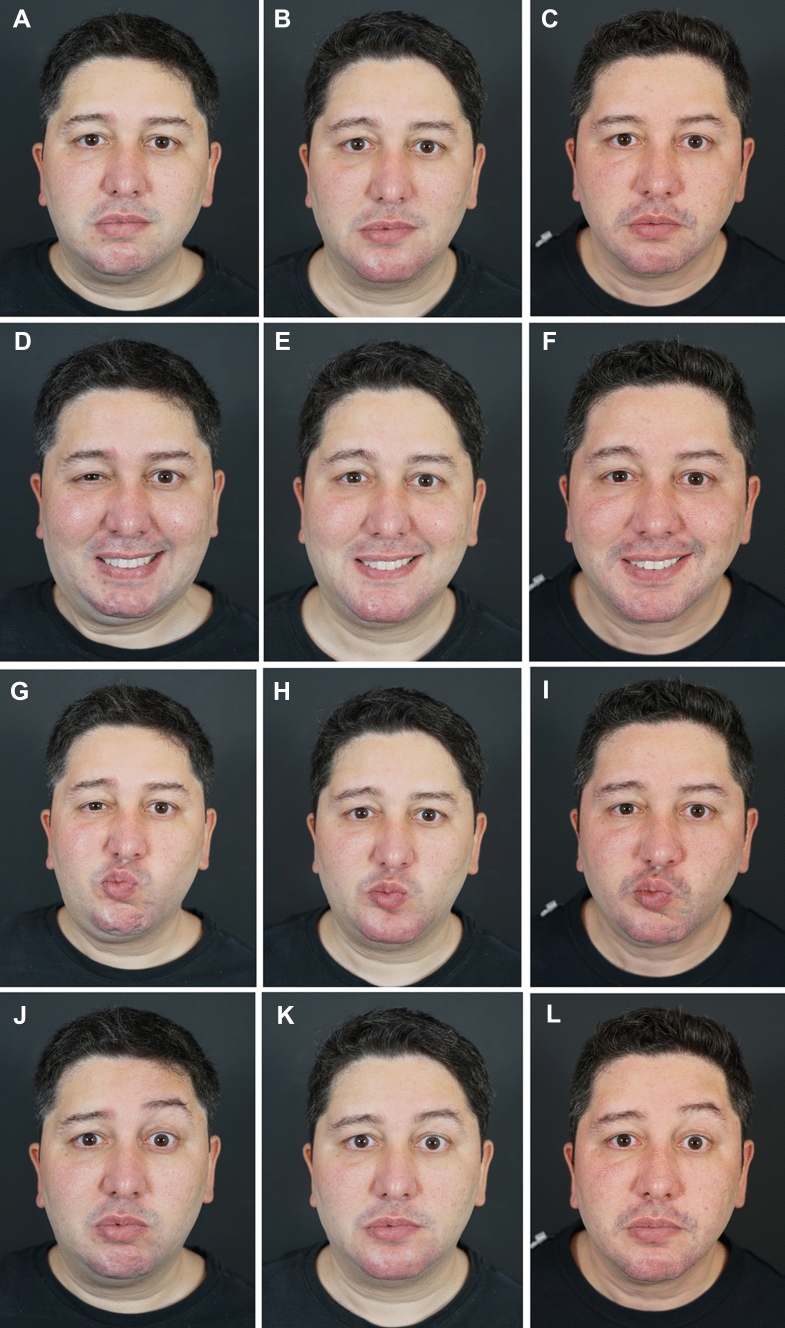

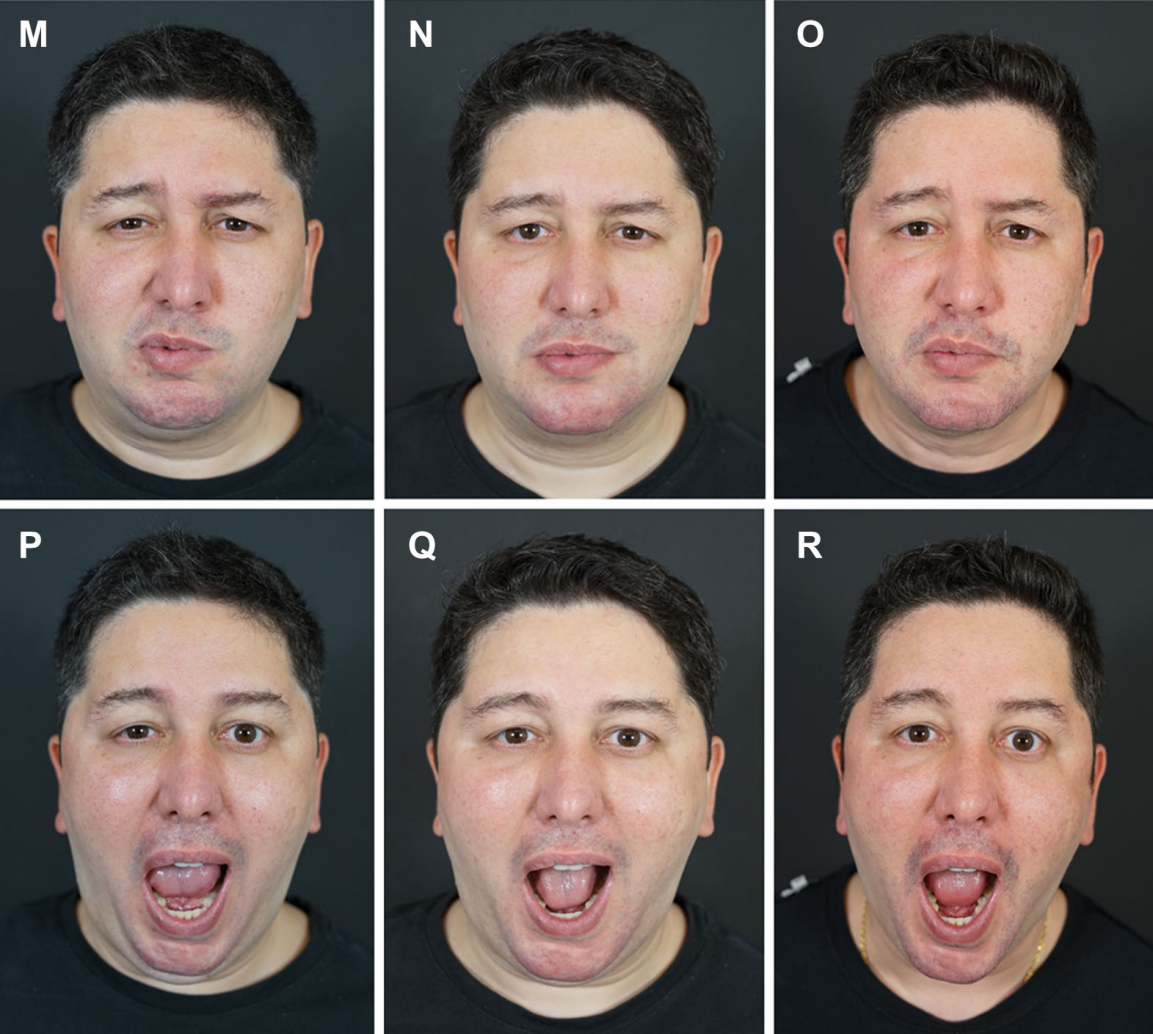
*Case Study 1.* (A, D, G, J, M, P) Pretreatment views of this 36-year-old man had been affected by facial paralysis, spasms, and synkinesis since age 16. He was treated with hyaluronic acid (HA) fillers to yield myomodulation. The total treatment volumes on the left and right sides of the face were 3.2 mL and 6.8 mL, respectively. (B, E, H, K, N, Q) Thirty days post-treatment. Note the reduced oral-ocular synkinesis, decreased dimpling of the chin, improved symmetry of the eyebrows, and more relaxed countenance overall. (C, F, I, L, O, R) Fifteen months post-treatment, the effect is durable. (Note that the patient lost considerable weight during this time.) Treatment of this patient and dynamic treatment outcomes are depicted in Supplemental Videos 1 and 2.

**Fig 4:**
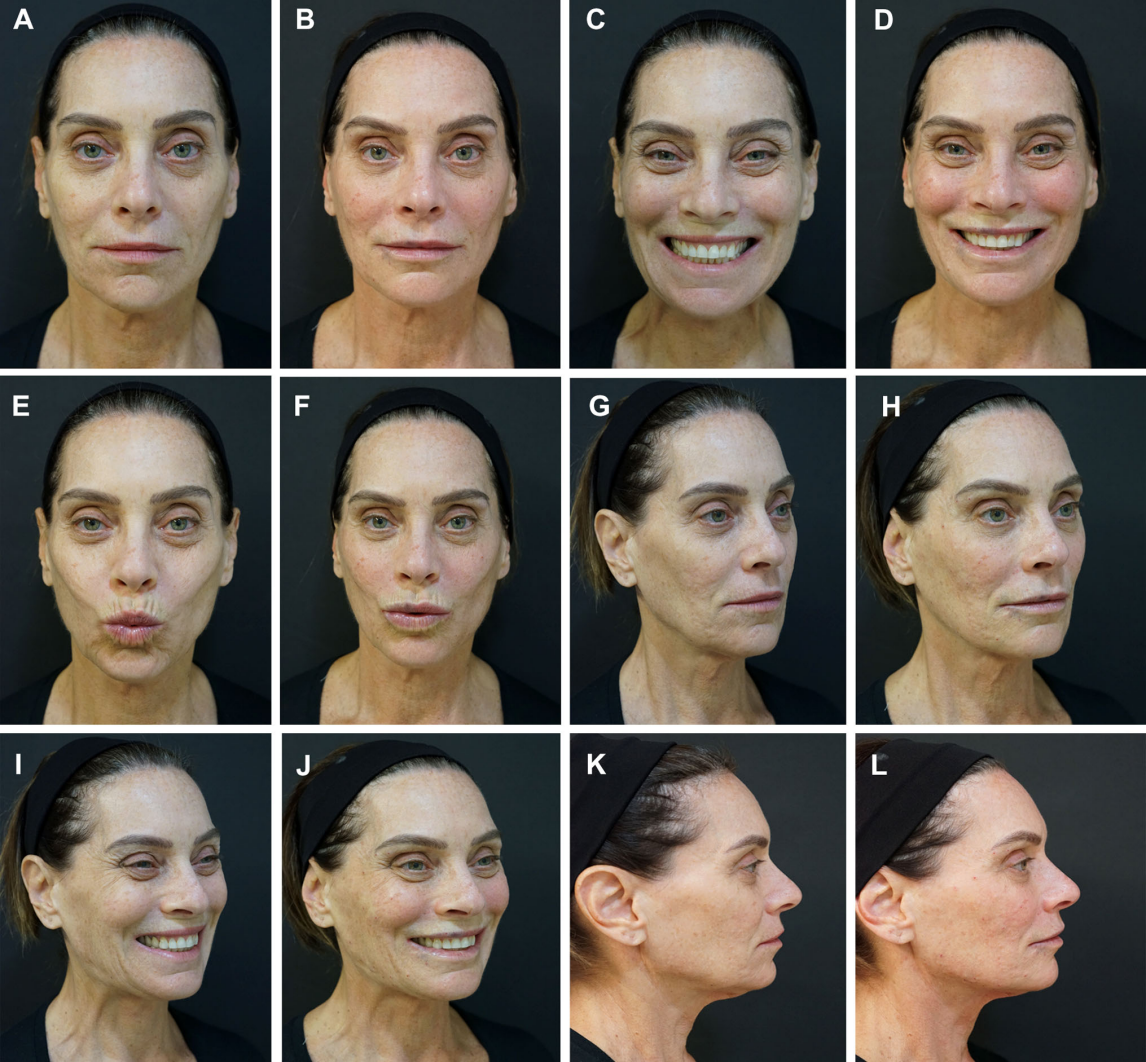
*Case Study 2.* **A**, **C**, **E**, **G**, **I**, **K** Pretreatment views of this 65-year-old woman who presented with cosmetic concerns related to aging and underwent myomodulation with HA fillers. The total volumes injected on the left and right sides of the face were 6.1 mL and 7.2 mL, respectively. **B**, **D**, **F**, **H**, **J**, **L** Immediately post-treatment. Note that the patient shows less effort on smiling, the eyes open more widely, and there is less contraction of the lateral platysma bands in the frontal view. In the oblique view, the patient looks more expressive at rest, with less visible jowls. On smiling, there is diminished age-related centralization of muscle activity, and she appears relaxed. In profile, the patient’s malar projection and jawline are aesthetically pleasing and do not appear overfilled. Treatment of this patient and dynamic treatment outcomes are depicted in Supplemental Videos 3 and 4.

The treatment procedures and treatment outcomes of 2 cases are depicted in Figures 3 and 4 and in Supplemental Videos 1–4.

## Discussion

Age-related bone loss and volume changes in the facial fat compartments yield ptosis of the lower third of the face and affect the ways in which the mimetic and sphincter muscle groups contract to form facial expressions. As the opposing levators and depressors shift caudally, other facial muscles become involved, particularly those of the midface. Consequently, muscle activity converges toward the nose and inner canthi of the eyes on smiling, and the height-to-width ratio of the eyes decreases. The authors describe this age-related phenomenon as the “centralization of facial muscle activity.”

Facial restoration techniques must account for the complex pattern of adipose and osseous tissue loss that occurs with age [8, 9]. Multiple authors have emphasized the importance of lateral filler placement (e.g., in the superior temples) to restore volume lost with aging [5, 6, 10, 11]. Using 3DD Lift, filler placement in the lateral subunits (1A, 1B, 1C, 3A, 5A) produces a lifting effect and facilitates levator action. This effect is evident with treatment of area 1A alone and is successively enhanced with treatment of the other subunits. Restoring structural support to the lateral face reduces ptosis of the anterior face, making it easier for the muscles in this highly mobile area of the face to contract. Treatment of these areas also tends to increase smile width (presumably by facilitating contraction of zygomaticus major and minor and the risorius) and diminishes age-related centralization of muscle activity, making smiling look effortless [5]. These results of filler injection seem to reflect both myomodulation of the treated subunit and myomodulatory effects in areas distal to the treatment site. [5]

In areas 2A, 4B, 6A, 7A, and 7B, structural support is provided by the maxilla, and the forehead and chin are essential for executing facial expressions. de Maio [3] proposed that management of the lateral SOOF with filler placed on the periosteum can engage the zygomaticus major like a pulley, thereby lifting the corners of the mouth on smiling. Consistent with this, deep injection onto the periosteum in the pyriform fossa (6A) appears to facilitate the LLS and LLSAN. Injecting filler into the subcutaneous layer of area 4C and the body of the mandible (5A) improves contour of the lower third of the face and diminishes unwanted depressor action of the DLI, DAO, and platysma. This also appears to facilitate contractility of the antagonist levators.

In the areas corresponding to the orbicularis oculi and orbicularis oris (2B, 3B, 4A, and 6B), volume restoration has the effect of making muscle contraction more difficult. The progressive loss of osseus and adipose structural support in these sphincter areas, together with skin laxity, contribute to rhytids around the eyes and mouth, even when the face is at rest. Filler placed in these subunits stretches the muscle and yields deep structural support, thereby reducing muscle spasticity and resetting the tonus. Similarly, restoration of volume in the chin by means of filler placement at the periosteal level helps reduce involuntary contraction of the mentalis and the peau d’orange appearance of the skin. Secondary to treatment with biostimulators, the increased collagen also improves tension of the skin, yielding a more youthful appearance at rest and on animation.

Myomodulation resulting from treatment of area 3B helps restore the resting tone of the occipitofrontal muscles and decreases synkinesis in the lower third of the face in patients with sequelae of facial paralysis; a representative case is shown in Supplemental Videos 1 and 2. In mouth-breathing patients and those with facial paralysis, filler placement in area 4A (mentolabial angle) has the dual effects of hindering contraction of the perioral sphincter muscle and improving lip incompetence. Because treatment of this subunit also restricts contraction of the mentalis, there is reduced need to engage the platysma when smiling.

Owing to a lack of excess skin, the forehead undergoes a different aging process from the lower face, with the lateral brow descending in the patient’s 40s or 50s, the inner brow remaining essentially in the same position, and the medial brow becoming elevated [12]. Horizontal rhytids in this region arise from the regression of bone and connective tissue supports. Adding volume to the loose areolar tissue below the galea aponeurotica restores convexity to this region and smooths these lines. [12]

Video depictions of patients on animation before and after myomodulation are crucial for allowing others to critique the outcomes of treatment. When viewed as static photographs, the altered muscle activity produced by myomodulation can make it seem as if the patient has made a behavioral modification or is exerting less effort. [12]

Interestingly, the authors have found that myomodulation of one functional group appears to affect the action of other mimetic muscle groups. In 10 years of performing myomodulatory treatment with facial fillers, this effect has been observed particularly in patients with sequelae of facial paralysis, in which the periocular treatment promotes improvements in chin and perioral dynamics and vice versa. Additionally, filler injection to influence the levator muscle of the chin not only affects muscle action in lifting the lower lip but also facilitates muscles affecting the cheek and eyebrow. Although speculative, this might be attributable to the stimulation of cutaneous mechanoreceptors and a corresponding effect on sympathetic outflow; such a phenomenon was demonstrated to occur between the hand/foot and the skeletal muscles [13]. Nevertheless, a limitation of the current study is that the empirical findings are contextualized in an unproven theory regarding the response of the mimetic and sphincter muscles to deposits of filler. Electrophysiologic studies are warranted to better understand the mechanistic basis of myomodulation.

It is worthwhile to compare our technique and results of myomodulation with those of other facial rejuvenation procedures. Marten and Elyassnia [14] present excellent results of facial fat grafting as a means of treating atrophy in conjunction with correction of ptosis by facelift. The authors assert that any areas treatable with injectable non-autologous fillers are eligible for fat injection [14]. However, they caution that the benefit of adipose tissue transfer is highly dependent on the patient also receiving facelift to ensure redundant tissue is excised. Fat transfer alone tends to produce overfilling that is difficult to subsequently correct [14]. Although the results of combined facelift and filler placement are superior to those of filler alone, our 3DD Lift technique takes advantage of the facial musculature to yield a noticeably lifted and contoured face without the added risks and recovery time of tissue resection. Like facial fat grafting, myomodulation with hyaluronic acid also requires caution to avoid an overfilled appearance, but we have found that our sequential lateral-to-medial approach is effective at avoiding this unfavorable outcome.

Over time, filler resorption and waning of the effect are possible with autologous and non-autologous fillers. Marten and Elyassnia [14] discuss the uncertainty of fat-graft take, and they note that atrophy after facial fat grafting tends to be distributed pan-facially. For this reason, facial fat grafting should involve multiple areas of the face for a harmonious result. Similarly, we typically address multiple facial subunits in any patient who undergoes 3DD Lift. In our experience, patients who present with muscle overactivity (e.g., spasticity or blepharospasm) tend to have slightly increased filler resorption in these areas. Nevertheless, as we demonstrate with Case Study 1 (see Figure 3), the effects of our myomodulatory technique are durable for well over 1 year, even in patients with long-standing facial paralysis and synkinesis.

## Conclusions

De Maio [3] asserted that a harmonious and attractive appearance can be achieved not by treating isolated problem areas but by considering the negative emotional countenance of the patient holistically (e.g., appearing sad, angry, or tired when the patient is not) and correcting this discordance with numerous filler boluses placed systematically throughout the face. The 3DD Lift technique involves strategic, sequential placement of HA filler relative to specific mimetic muscles to influence muscle action.^1, 6^ Although myomodulation with injectable fillers is a relatively new practice, the clinical results have been promising. Additional studies are warranted to investigate the mechanism of myomodulation and to assess reproducibility of these techniques among practitioners.

## Supplementary Information

Below is the link to the electronic supplementary material.

Supplementary file1 (MP4 333239 kb)

Supplementary file2 (MP4 269252 kb)

Supplementary file3 (MP4 303203 kb)

Supplementary file4 (MP4 168581 kb)
